# Supplementary material for: A Qualitative Evaluation of the Barriers and Enablers for Implementation of an Asymptomatic SARS-CoV-2 Testing Service at the University of Nottingham: A Multi-Site Higher Education Setting in England
Source: Int J Environ Res Public Health. 2022 Oct 12;19(20):13140. doi: 10.3390/ijerph192013140 (PMC9603241; doi:10.3390/ijerph192013140)
Supplement: Supplementary file 1 [file ijerph-19-13140-s001.zip › S1_Interview Topic Guide.pdf]

Supplementary file 1
ATS Staff Interview Topic Guide

Your views towards the ATS

1. To what extent do you think that mass testing was helpful in containing the spread of COVID-19 in universities? What impact (if any) do you think the service will have had on containing the spread of COVID-19 (in the short-term, medium-term, and longer-term)?

2. What has the overall impact of the service been on students?

3. What has the overall impact of the service been on staff?

4. Overall, what worked well in the ATS? What were the particular features of the ATS and the local or national context that were most helpful / made a positive difference?

5. Overall, what worked less well? What were the particular features of the ATS and the local or national context that were least helpful / had a negative impact?

6. Was there anything else that influenced *how* the ATS was implemented as a service, and the outcome?

7. What is your view on the University having used resources to support the service?

8. Anything else you would like to tell us? Recommendations for the future?
