# Supplementary material for: A Qualitative Evaluation of the Barriers and Enablers for Implementation of an Asymptomatic SARS-CoV-2 Testing Service at the University of Nottingham: A Multi-Site Higher Education Setting in England
Source: Int J Environ Res Public Health. 2022 Oct 12;19(20):13140. doi: 10.3390/ijerph192013140 (PMC9603241; doi:10.3390/ijerph192013140)
Supplement: Supplementary file 1 [file ijerph-19-13140-s001.zip › S2_Participant characteristics.pdf]

Table S1. Participant characteristics.

Respondent	Gender[a]	Age	Job role[b]
1	F	20-30	Research
2	F	20-30	Research
3	M	31-40	Laboratory
4	F	61-70	APM[c]
5	F	31-40	APM
6	F	61-70	Laboratory
7	M	31-40	Laboratory
8	M	41-50	Research
9	F	31-40	Laboratory
10	M	21-30	Laboratory
11	F	31-40	Laboratory
12	M	41-50	Research
13	F	51-60	APM
14	F	20-30	APM
15	F	31-40	Laboratory
16	F	51-60	APM
17	M	31-40	APM
18	F	51-60	Laboratory
19	F	20-30	Research
20	F	41-50	Research
21	F	20-30	APM
22	F	61-70	APM
23	M	61-70	Research
24	M	31-40	Other[d]
25	M	41-50	APM

[a]F=female, M=male; [b]prior to joining the ATS; [c]Administrative, professional and managerial; [d]Other: IT or technical.
